# Fused behavior recognition model based on attention mechanism

**DOI:** 10.1186/s42492-020-00045-x

**Published:** 2020-03-12

**Authors:** Lei Chen, Rui Liu, Dongsheng Zhou, Xin Yang, Qiang Zhang

**Affiliations:** 1 Key Laboratory of Advanced Design and Intelligent Computing, Ministry of Education, School of Software, Dalian University, Dalian, 116622 China; 2 School of Computer Science and Technology, Dalian University of Technology, Dalian, 116024 China

**Keywords:** Action recognition, ResNet34-3DRes18, Res34-SE-IM-net, Attention mechanism

## Abstract

With the rapid development of deep learning technology, behavior recognition based on video streams has made great progress in recent years. However, there are also some problems that must be solved: (1) In order to improve behavior recognition performance, the models have tended to become deeper, wider, and more complex. However, some new problems have been introduced also, such as that their real-time performance decreases; (2) Some actions in existing datasets are so similar that they are difficult to distinguish. To solve these problems, the ResNet34-3DRes18 model, which is a lightweight and efficient two-dimensional (2D) and three-dimensional (3D) fused model, is constructed in this study. The model used 2D convolutional neural network (2DCNN) to obtain the feature maps of input images and 3D convolutional neural network (3DCNN) to process the temporal relationships between frames, which made the model not only make use of 3DCNN’s advantages on video temporal modeling but reduced model complexity. Compared with state-of-the-art models, this method has shown excellent performance at a faster speed. Furthermore, to distinguish between similar motions in the datasets, an attention gate mechanism is added, and a Res34-SE-IM-Net attention recognition model is constructed. The Res34-SE-IM-Net achieved 71.85%, 92.196%, and 36.5% top-1 accuracy (The predicting label obtained from model is the largest one in the output probability vector. If the label is the same as the target label of the motion, the classification is correct.) respectively on the test sets of the HMDB51, UCF101, and Something-Something v1 datasets.

## Introduction

Human behavior recognition based on video streams has been widely used in security monitoring, human-computer interaction, and automatic driving, etc. It has attracted the attention of many scholars and research institutions. With the rapid development of deep learning technology, many great achievements have been obtained for behavior recognition tasks in recent years.

**Fig. 1:**
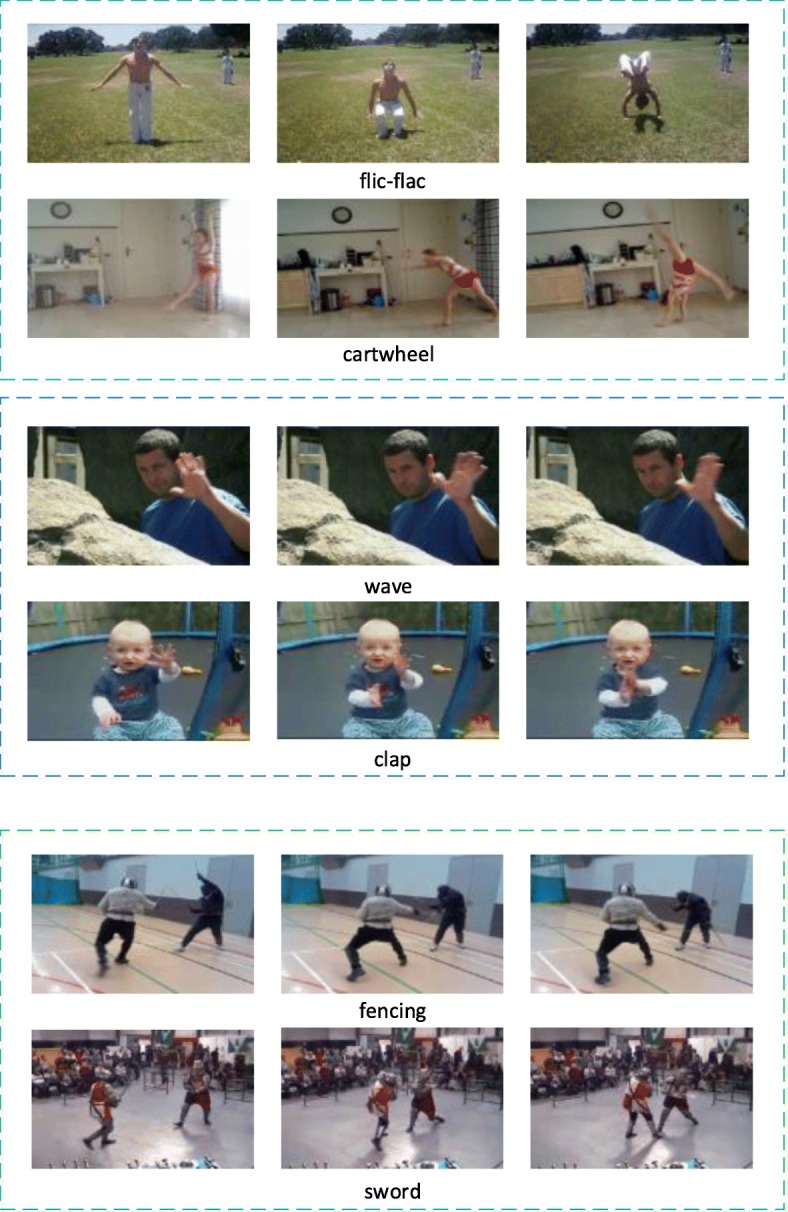
Some actions easily confused with each other in datasets

However, there are still problems that must be solved for the behavior recognition task: There are many confusing actions in the existing datasets, which affect the performance of the models. In order to train efficient behavior recognition models, researchers have built many video datasets, such as HMDB51 [1], UCF101 [2], Kinetics [3], Something-Something v1 [4], etc. Some of the actions in these datasets are easily confused with one another, such as ‘flic-flac’ and ‘cartwheel’, ‘wave’ and ‘clap’, ‘fencing’ and ‘sword’, as shown in Fig. 1.

Considering the advantages of the two-dimensional (2D) and three-dimensional (3D) fused model in practical application, we constructed a 2D and 3D fused network, ResNet34-3DRes18, as our baseline model in this study. The network was relatively lighter and achieved results comparable with those of state-of-the-art network. To make the model better able to distinguish between confusing actions, an attention mechanism was added to the basic ResNet34-3DRes18 model, creating a new model named Res34-SE-IM-Net. This method could distinguish between confusing motions effectively in the existing behavior recognition datasets (in this study, HMDB51 was used as an example), which thus improved the overall accuracy of the model to a certain extent. The remaining sections of this paper are organized as follows. First, the related work about video behavior recognition is introduced in the second section. Then, the behavior recognition models proposed in this study are described in the third section. The performances of our methods on different datasets are then evaluated in the fourth section; Finally, the paper is summarized in the fifth section.

The main contributions of this paper include the following.

- We proposed an efficient 2D and 3D fused video behavior recognition model which acquired good performance on some challenging datasets and had faster video processing speed (VPS).
- We proposed a video behavior recognition model based on an attention mechanism, named Res34-SE-IM-Net. This method can effectively distinguish between confusing actions and therefore improved model performance.

## Related work

Convolutional neural networks (CNNs) have gradually replaced traditional behavior recognition methods which are characterized by manual feature extractors. Methods based on CNNs have become mainstream methods for behavior recognition. Based on different convolutional kernels, these methods can be divided into 2D CNNs, 3D CNNs and 2D and 3D fused networks.

With the great success of CNNs in the field of image classification, the transfer CNNs from image classification to behavior recognition has attracted considerable attention from researchers. Simonyan and Zisserman [5] proposed a two-stream network that consisted of a spatial stream and a temporal stream. Of these, the spatial stream was used to extract spatial features, and the temporal stream learned the temporal relationships between frames. Feichtenhofer et al. [6] improved the two-stream network by fusing the two branches in the convolutional layer instead of using late fusion. Wang et al. [7] adopted a video segmentation sampling strategy to obtain the input for the network, so that the two-stream network could make full use of the information in the entire video. Although these methods achieved good results, they did not achieve good performance on complex temporal modeling problems.

One straightforward and effective way to solve the complex temporal modeling problem with videos is to expand the 2D convolution kernel to a 3D convolution kernel to build a 3D CNN network. Tran et al. [8] proposed a C3D model, which demonstrated that a 3D CNN is better at learning spatiotemporal features than a 2D CNN. Carreira and Zisserman [9] proposed a deeper 3D CNN named I3D, which achieved better results than C3D on existing behavior recognition datasets. However, 3D CNNs usually have a complex structure, for example the C3D network. This network had only 11 layers [10], but its model size was much larger than the deeper 2D CNN networks.

In order to reduce the complexity of models and ensure their performance, researchers have tried to construct a behavior recognition model by fusing 2D CNNs and 3D CNNs [11]. Zolfaghari et al. [12] constructed an online action recognition model using a 2D and 3D fused model. This type of network achieves an accuracy comparable with the state-of-the-art networks at a faster speed.

## Methods

In this study, two behavior recognition models are proposed, the ResNet34-3DRes18 network and the Res34-SE-IM-Net model. We first introduce the basic structure of the two models, and then elaborate on the specific details of the models.

### ResNet34-3DRes18

**Fig. 2:**
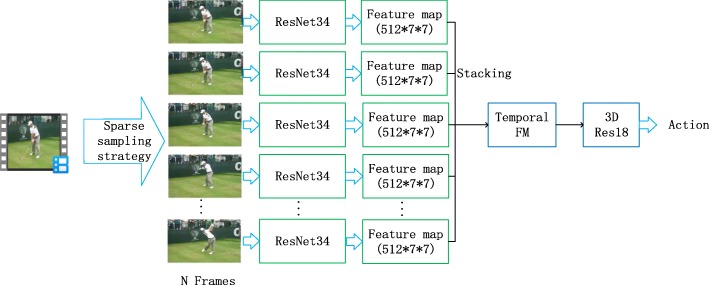
A architecture of ResNet34-3DRes18 network. The N frames images are obtained by the sparse sampling strategy. Then these images are processed by ResNet34 network to get their Feature map. The Feature map are stacked to obtain a temporal feature map, named Temporal FM. The Temporal FM is processed by 3DRes18 network to get the final action recognition result

A fused network with a 2D CNN and a 3D CNN, named ResNet34-3DRes18, was first designed. Its architecture is a typical top-heavy hybrid network [13] with both 2D and 3D characteristics, as shown in Fig. 2. Specifically, the spatial features of a single image are first extracted by the 2D part of the CNN. The temporal relationships between different frames are then learned by the 3D CNN part. By processing this information jointly, we obtain get the final action class label.

According to ref. [10], shallower networks cannot achieve better results than the deeper networks. However, deeper networks also have disadvantages, such as a large number of parameters and greater requirements for hardware configuration. Considering the performance and complexity of the network, the modified ResNet34 [14] network (until layer 4) was selected as the 2D CNN part which obtains the spatial features of the input images. For the 3D CNN part, the 3D-ResNet18 [15] network was used. The specific details for the 2D CNN and 3D CNN parts can be found in section 3.3.

The sparse sampling strategy used in ref. [12] was used to obtain N sampling frames as the input to the ResNet34 network. After processing by ResNet34 network, each input image was converted to 512 feature representation code maps. We named them as Feature maps. The size of each map is 7 × 7. This process can be shown in Function (1).

1 $$ {F}_{\mathrm{i}}={f}_{res34}\left({f}_i\right) $$

where *F*_*i*_ is a Feature map of the *i*_*th*_ sampling frame, *F*_*i*_ ∈ *R*^512 × 7 × 7^; the *f*_*res*34_ function represents the ResNet34 network, and *f*_*i*_ is the *i*_*th*_ sampling frame of the input video. Then, these Feature maps are stacked to obtain a feature map with a temporal dimension named Temporal FM, as shown in Function (2).

2 $$ TFM={f}_{stack}\left({F}_1,{F}_2,...,{F}_n\right) $$

where *TFM* is a temporal feature map, *TFM* ∈ *R*^512 × *N* × 7 × 7^; the *f*_*stack*_ is the stacking function for the feature maps; *n* is the number of input frames. The TFM is then sent to the 3DRes18 (3DCNN part) network for processing, and the results of the action recognition is finally obtained, as shown in Function (3).

3 $$ \left[ clas{s}_1, clas{s}_2,..., clas{s}_N\right]=\mathrm{softmax}\left({f}_{3D\operatorname{Re}s18}(TFM)\right) $$

where *class*_*i*_ is the probability value that an input video belongs to the *i*_*th*_ class; *N* is the total number of classes; the ‘softmax’ represents the normalized function and the *f*_3*D* Re *s*18_ is the 3D-Res18 network. The final action label is the index class of *class*_*i*_, which has the max probability value in the vector of [*class*_1_, *class*_2_, ..., *class*_*N*_].

### Res34-SE-IM-net model

**Fig. 3:**
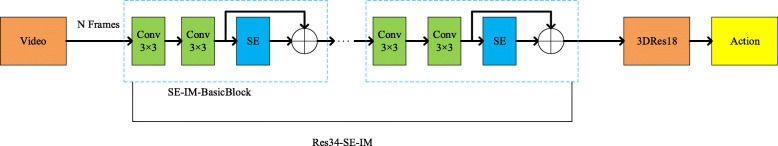
A architecture of Res34-SE-IM-Net network

Although the ResNet34-3DRes18 model takes full advantage of the 2D and 3D fused model, it is unable to distinguish between confusing motions. Because the model cannot distinguish the importance of different feature channels effectively, the method cannot focus on better distinguishinge information for a action. The squeeze-and-excitation network [16] (SE Module) can explicitly model different feature channels, suppress useless feature channels, and enhance useful feature channels. Therefore, the SE Module can make model pay more attention to the feature channel which is more distinguished for the action recognition. Moreover, the module consumes only small amount of computation and basically not increase the complexity of models when modeling the feature channel. At the same time, the attention unit can be easily embedded into existing networks. Therefore, an SE Module and identity mapping [14] were introduced into the ResNet34-3DRes18 network, creating a new model that was named Res34-SE-IM-Net. This method is better able to distinguish between confusing actions than is the ResNet34-3DRes18 model. The architecture of the Res34-SE-IM-Net model is shown in Fig. 3. A video is first split into N frames images. And then these images are processed by the Res34-SE-IM network which consists of 16 SE-IM-BasicBlock, to get the feature maps of the images. Finally, these feature maps are stacking and then fed into 3DRes18 network to process. After this, the final action recognition results can be obtained.

**Fig. 4:**
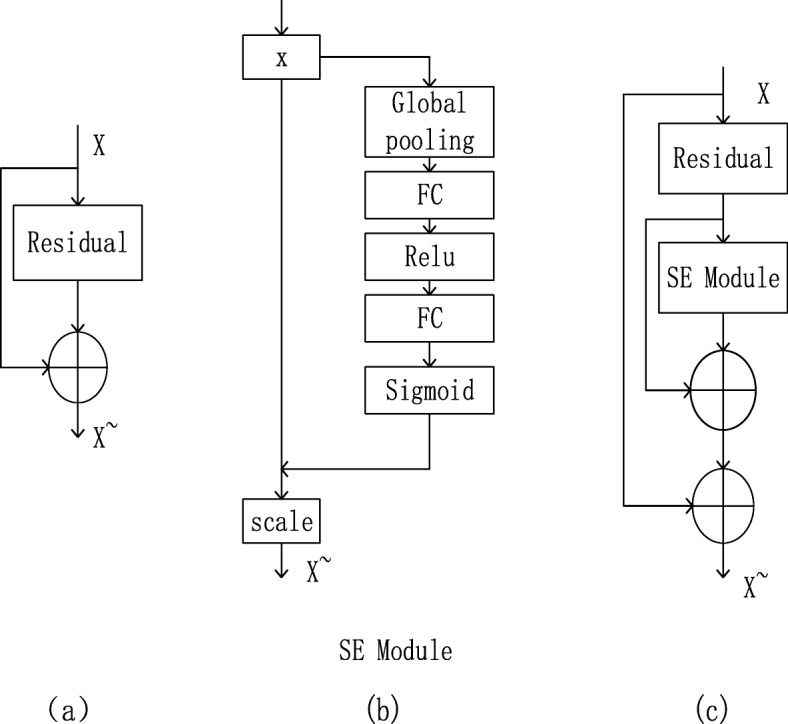
SE Module. (**a**) BasicBlock; (**b**) SE Module; (**c**) SE-IM-BasicBlock

The residual unit in ResNet, named BasicBlock, is shown in Fig. 4 (a). It can be seen from ref. [16] that the squeeze-and-excitation network is easily embedded into existing networks and achieves better results than the original network, as shown in Fig. 4 (b). Therefore, after the SE module is added, the model can focus more on the most distinguishing information for different actions. A residual attention unit, named SE-IM-BasicBlock, was constructed, as shown in Fig. 4 (c). This unit was easily embedded in the ResNet34 network, replacing the original residual block in the residual network, as shown in Function (4).

4 $$ {X}^{\sim }=X+\left[{f}_{resdiual}(X)+{f}_{SE}\left({f}_{resdiual}(X)\right)\right] $$

where *X* is the raw input of the network; *X*^~^ is the output of the SE-IM-BasicBlock; the *f*_*resdiual*_ function is the residual unit; the *f*_*SE*_ function is the SE Module.

### Details of network

#### ResNet34 network

For the 2D CNN part, the ResNet34network (up to layer 4) is used in this study. The network consisted of convolutional layers and pooling layers, as shown in Table 1. Of these, Layer1, Layer2, Layer3, and Layer4 represent different stages, which consist of different residual units. The residual units have different number of output channels, such as 64, 128, 256, and 512. Each residual unit (BasicBlock) consists of two 3 × 3 2D convolutions. After each input image is processed by the network, the image is converted to 512 feature representation code maps with a size of 7 × 7.

**Table 1:** Network architecture of ResNet34 and 3D Res18

ResNet34			3D Res18		
Layer name	Output size	The architecture of ResNet34	Layer name	Output size	The architecture of 3D Res18
Conv1	112 × 112	[2D conv7 × 7 64]	Conv1	7 × 7 × 128	{3Dconv3 × 3 × 3128 3Dconv3 × 3 × 3128} × 2
pool	56 × 56	[max pool 3 × 3]	Conv2	7 × 7 × 256	{3Dconv3 × 3 × 3256 3Dconv3 × 3 × 3256} × 2
Layer1	56 × 56	{2D conv3 × 3 64 2D conv3 × 3 64} × 3	Conv3	7 × 7 × 512	{3Dconv3 × 3 × 3512 3Dconv3 × 3 × 3512} × 2
Layer2	28 × 28	{2D conv3 × 3128 2D conv3 × 3128} × 4	Pooling	1 × 1 × 512	[Avgpool3D 1 × 7 × 7]
Layer3	14 × 14	{2D conv3 × 3256 2D conv3 × 3256} × 6	Dropout	1 × 8192	Dropout (*p* = 0.5)
Layer4	7 × 7	{2D conv3 × 3512 2D conv3 × 3512} × 3	–	1 × classes	FC, softmax

#### 3DRes18 network

For the 3D CNN part, the modified 3DResNet18 network was used to model the temporal relationships between different frames, as shown in Table 1. The network consists of convolutional layers, average pooling layers (Avgpool), a dropout layer, and a fully connected (FC) layer. In the convolutional layers, the convolutional kernel size is 3 × 3 × 3. These convolutional layers have different number of output channels, which are 128, 256, and 512. To reduce the risk of model over-fitting, a dropout layer of *p* = 0.5 is used. For the FC, the ‘classes’ represent the number of motion classes in the datasets.

## Results

First, the datasets and details of the experiment are introduced. Our methods are then evaluated on different action recognition datasets, which include the HMDB51, UCF101, and Something-Something v1 datasets. Compared with state-of-the-art methods, our methods showed better performance. At the same time, our methods required fewer parameters and showed higher processing speed, which makes them easy to deploy in practical applications. In addition, our approaches were also evaluated for the online classification task.

### Datasets and details of experiment

Our methods were evaluated on three different datasets, including the HMDB51, UCF101, and Something-Something v1 datasets. Of these, the HMDB51 and UCF101 are two popular datasets in the behavior recognition field that are usually used as standards for algorithm evaluation. The Something-Something v1 is a new, large human action dataset, that contains more than 100,000 motion clips and relies heavily on a temporal context. Thus, it is a challenging new dataset for behavior recognition. The details of these datasets are shown in Table 2.

**Table 2:** Details of HMDB51, UCF101 and Something-Something v1 datasets

Dataset name	Classes	Total clips	Clips/class
HMDB51	51	6766	102 (min)
UCF101	101	13,320	101 (min)
Something-Something v1	174	108,499	77–986

In the input part, the videos in the datasets were split into single frame images using the OpenCV library. After that, each video was divided into N segments of equal length, and one frame image was randomly selected from each segment as the input to the ResNet34 network. For the input images, data augmentation techniques, such as the fixed-corner cropping and scale jittering in ref. [12], were also applied to reduce the risk of model over-fitting.

The models were first pre-trained on the Kinetics database and then fine-tuned on the HMDB51, UCF101, and Something-Something v1 datasets. The hyper-parameters for the experiment are shown in Table 3. Sixteen frames of images (Num-segments = 16) in a video were chose as the input of our models. Batch-size represents the number of samples for training once. During the training, the initial learning rate (Lr) of the network was set to 0.001. When the accuracy of top-1 on the validation reached saturation for 5 consecutive epochs (Num-saturate = 5), the Lr automatically decreased by a factor of 10. In order to avoid gradient explosion during training, a gradient threshold (clip-gradient = 50) was set. In our model, the Stochastic Gradient Descent optimizer with Momentum decay (helping accelerate gradient update) and Weight-decay (a measure of reducing over-fitting) was used. At the same time, a dropout layer (Dropout = 0.5) was applied before the FC in order to prevent the model from over-fitting.

**Table 3:** The critical hyper-parameters of the experiment

Num-segments (N)	16	Dropout	0.5
Batch-size	16	clip-gradient	50
Lr	0.001	Momentum	0.9
Weight-decay	5e-4	Num-saturate	5

### Comparison to state-of-the-art methods on different datasets

To evaluate the performance of our methods, we compared them with state-of-the-art methods, as shown in Tables 4 and 5. On the HMDB51 and UCF101 datasets, our approaches are compared with the traditional methods (in the first row), the deep learning methods using RGB as input (in the second row), and the deep learning methods using multimodal input (in the third row), as shown in Table 4. On the HMDB51 and UCF101 datasets, all our methods achieved better performance except for I3D, which used a deeper network. Our methods also attained better performance on the Something-Something v1 dataset, even though this dataset is more complicated and depends heavily on temporal relationships.

**Table 4:** Comparison of recognition accuracy with state-of-the-art methods on HMDB51 and UCF101 datasets

Methods	Input modality	Pre_training	HMDB51 (%)	UCF101 (%)
HOG/HOF [1]	RGB	–	20.44	–
IDT [17]	RGB	–	57.2	85.9
MIFS [18]	RGB	–	65.1	89.1
ECO-Lite (16 frames) [12]	RGB	Kinetics	68.2	91.6
ECO (16 frames) [12]	RGB	Kinetics	68.5	92.8
ResNext-101 [19]	RGB	Kinetics	63.8	90.7
Res3D [15]	RGB	Sports-1 M	54.9	85.8
I3D [9]	RGB	Kinetics	74.5	95.4
ResNet101 [19]	RGB	Kinetics	61.7	88.9
DTTP (split 1) [20]	RGB	ImageNet	61.5	89.7
RSN [21]	RGB	–	55.9	87.5
Two-stream (fusion by SVM) [5]	RGB, Optical flow	ILSVRC	59.4	88.0
VGG16 + TSN [22]	RGB,Optical flow	ImageNet	67.3	92.1
ResNet34-3DRes18 (16 frames)	RGB	Kinetics	70.997	92.143
Res34-SE-IM-Net (16 frames)	RGB	Kinetics	71.85	92.196

**Table 5:** Comparison of recognition accuracy with state-of-the-art methods on Something-Something v1 dataset

Methods	Input modality	Pre_training	Top-1 val (%)	TOP-1test (%)
TSN by ref. [23] (7 frames)	RGB	ImageNet	18.48	–
MultiScale TRN [23]	RGB	ImageNet	34.44	33.6
ECO (16 frames) [12]	RGB	ImageNet	41.4	–
TRN (ResNet-50) by ref. [13] (8frames)	RGB	ImageNet	38.9	–
ResNet34-3DRes18 (16 frames)	RGB	Kinetics	41.012	–
Res34-SE-IM-Net (16 frames)	RGB	Kinetics	41.398	36.5

### Complexity and accuracy comparison

In order to demonstrate our methods (ResNet34-3DRes18 and Res34-SE-IM-Net) lighter and more effective than other approaches, some relational indicators that evaluate the complexity and accuracy of models are listed in Table 6. The number of floating point operations (FLOPs) represents the number of floating-point operations, which can precisely measure the complexity of models; The models’ parameters (Param) indicate the number of model parameters, such as weight and bias; The number of model’s layers (Depth) denotes the number of model layers which not include the ‘BN’ layers; ‘VPS’ represents the number of videos processed per second. We can see clearly from Table 6 that our methods acquire better performance at the expense of a shallower network, lower FLOPs, and higher VPS, except I3D. I3D has a lesser number of parameters because it uses many smaller convolutional kernels, such as 1 × 1 × 1. However, it has a larger FLOPs and a lower VPS, which makes the method difficult to employ in the practical applications. The depth of Res34-SE-IM-Net does not include the two linear layers in the SE Module, because the linear layers have fewer parameters.

**Table 6:** Comparison of the complexity and accuracy between our methods and state-of-the-art methods on the HMDB51 and UCF101 datasets

Methods	FLOPs	Param	Depth	VPS	HMDB51 (%)	UCF101 (%)
I3D(RGB) [9]	139.39G	12.7 M	72	0.5	74.5	95.4
ResNext-101 [19]	192.31G	60.63 M	101	–	63.8	90.7
ResNet-101 [19]	277.23G	86.92 M	101	–	61.7	88.9
ResNet34-3DRes18 (16 frames)	85.57G	55.78 M	48	20.2	70.997	92.143
Res34-SE-IM-Net (16 frames)	85.6G	60.2 M	48	18.8	71.85	92.196

### ResNet34-3DRes18 and Res34-SE-IM-net

ResNet34-3DRes18 and Res34-SE-IM-Net were respectively evaluated on the test set of HMDB51 and UCF101, and the validation set of Something-Something v1, as shown in Table 7. It can be seen that Res34-SE-IM-Net achieved better performance on the three datasets, than did ResNet34-3DRes18.This fully proves the effectiveness of introducing the SE module and identity mapping in the basic ResNet34-3DRes18. Furthermore, compared with ResNet34-3DRes18, the FLOPs and Param of Res34-SE-IM-Net model were increased very little, which was compensated for its superior performance, as shown in Table 6.

**Table 7:** Comparison of recognition accuracy between ResNet34-3DRes18 and Res34-SE-IM-Net on HMDB51, UCF101 and Something-Something v1 datasets

Dataset	Methods	Top-1 (%)	Top-5 (%)
HMDB51(test set)	ResNet34-3DRes18	70.997	90.748
	Res34-SE-IM-Net	71.85 (+ 0.853)	91.535 (+ 0.787)
UCF101(test set)	ResNet34-3DRes18	92.143	99.392
	Res34-SE-IM-Net	92.196 (+ 0.053)	98.862
Something-Something v1(validation set)	ResNet34-3DRes18	41.012	72.139
	Res34-SE-IM-Net	41.398 (+ 0.386)	72.743 (+ 0.604)

To further demonstrate the advantages of Res34-SE-IM-Net in distinguishing confusing motions, we introduce a confusion indicator [24] to evaluate its performance. Confusion refers to the sum of the probability that two different motions will be misidentified as the other. Owing to the better performance of our methods on the HMDB51 dataset, we chose to use it as an example to illustrate the problem. The confusion of some actions on our methods are compared, as shown in Table 8. The two kinds of movements enclosed in parentheses are easily confused movements such as “(flic-flac, cartwheel)”, and the values below it indicate the confusion between them. We can see clearly that the Res34-SE-IM-Net model acieved lower confusion for most of the confusing actions than did the ResNet34-3DRes18 model. This fully demonstrated that the Res34-SE-IM-Net was better able to distinguish between confusing actions than was ResNet34-3DRes18.

**Table 8:** Comparison of the confusion between ResNet34-3DRes18 and Res34-SE-IM-Net

Confusing actions	ResNet34-3DRes18 (16frames)	Res34-SE-IM-Net (16 frames)
(flic-flac, cartwheel)	43%	30% (−13%)
(wave, clap)	32%	11% (−21%)
(laugh, smile)	37%	16% (−21%)
(fencing, sword)	40%	37% (−3%)
(cartwheel, handstand)	26%	24% (−2%)

### Online recognition

**Fig. 5:**
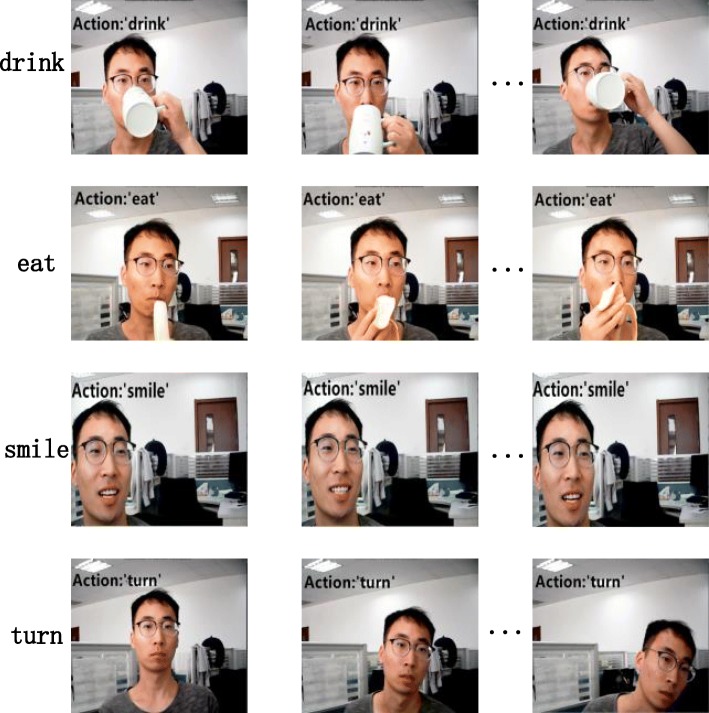
Results of online recognition of the Res34-SE-IM-Net network

To verify the performance of our method in practical application, we used the Res34-SE-IM-Net model in an online action recognition task. The input of the network was obtained using a GUCEE HD98 digital camera. After capturing the input videos, the input of the Res34-SE-IM-Net model was obtained using the online sampling strategy in ref. [12]. The input was then sent to the model to obtain real-time action recognition results. The results for the online recognition of the Res34-SE-IM-Net model are shown in Fig. 5. Each line represents the recognition result of one class of motion. The text to the left of the images indicates the true categories of the input actions, while the black text on the images indicates the predicted categories of these actions. As can be seen from the figure, the Res34-SE-IM-Net model can accurately distinguish between confusing motions (such as ‘drink’ and ‘eat’) under real-time conditions, and obtains good results in real-world applications.

## Conclusions

In this study, we proposed an improved 2D and 3D fused video behavior recognition model named ResNet34-3DRes18. The model is composed of 2DCNN part (ResNet34) and 3DCNN part (3DRes18). This method attained better performance with higher speed than state-of-the-art methods. Furthermore, in order to strengthen the ability of the model to distinguish between easily confused motions, the SE Module and identity mapping are introduced into the ResNet34-3DRes18 network, constructing the Res34-SE-IM-Net network. The model achieved better performance on the HMDB51, UCF101, and Something-Something v1 datasets, than did the ResNet34-3DRes18 network. Our method showed better results on the online action classification task.

Although the Res34-SE-IM-Net network can distinguish some confusing motions to some extent, the model can’t effectively model the complex temporal motions such as some actions in Something-Something v1 dataset. Therefore, in our future work, we will consider designing some temporal attention modules and adding them to the model to increase the model’s discrimination of different frames in a video.

## Data Availability

The datasets used or analyzed during current study are public available.

## Abbreviations

2D: Two-dimensional
2DCNN: 2D convolutional neural network
3D: Three-dimensional
3DCNN: 3D convolutional neural network
clip-gradient: Gradient threshold
CNN: Convolutional neural network
Depth: The number of model’s layers
FC: Fully connected
FLOPs: The number of floating point operations
Lr: Learning rate
Param: The models’ parameters
SE Module: Squeeze-and-excitation network
VPS: The numbers of processed videos per second
VPS: Video processing speed
